# Multicomponent Interventions for Adults With Cancer Cachexia: A Systematic Review

**DOI:** 10.1002/jcsm.13716

**Published:** 2025-02-27

**Authors:** Megan Bowers, Carmine Petrasso, Amy McLuskie, Joanne Bayly, Barry J. A. Laird, Irene J. Higginson, Matthew Maddocks

**Affiliations:** ^1^ Cicely Saunders Institute of Palliative Care, Policy & Rehabilitation, Florence Nightingale Faculty of Nursing, Midwifery & Palliative Care King's College London London UK; ^2^ Edinburgh Palliative Care and Supportive Care Group The University of Edinburgh Edinburgh UK; ^3^ St Columba's Hospice Edinburgh UK

**Keywords:** cachexia, cancer, multimodal, quality of life, treatment, weight loss

## Abstract

**Background:**

Cancer cachexia has substantial impacts on people's quality of life. There is no current gold standard treatment, but the complex pathophysiology of cachexia suggests that a multitargeted and individualised treatment approach is needed. We aimed to evaluate the extent to which multicomponent interventions have targeted the key features of cachexia and been tailored to individuals, and differential effects on quality of life.

**Methods:**

We conducted a systematic review of multicomponent interventions for adults with cancer cachexia. We searched four databases, two clinical trial registers and MedRxiv on 20 June 2024. Intervention components were classified by intervention category (nutritional, exercise/physical activity, pharmacological and psychosocial), cachexia feature(s) targeted (reduced energy intake, altered metabolism, involuntary weight loss and decline in physical function) and level of tailoring. Within‐arm standardised mean changes in quality of life over time, as well as standardised mean differences between study arms, were calculated.

**Results:**

Sixty‐two multicomponent interventions were included, of which two combined components from all four intervention categories, and nine targeted all four key features of cachexia. Eighteen multicomponent interventions were fully tailored and 30 were partly tailored to individuals. Within‐arm standardised mean changes in quality of life were calculated for thirteen studies; all had a high risk of bias or raised concerns. In eleven studies, quality of life scores improved following the intervention, whereas in two studies they declined. Standardised mean differences between study arms were calculated for four studies; in three, the intervention arm showed a greater improvement in quality of life scores than the usual care arm. Amongst these data, there was no indication that the number of cachexia features targeted, or the extent of tailoring, was associated with a greater improvement in quality of life scores; however, the heterogeneity prevented us from concluding on our hypothesis.

**Conclusions:**

This review mapped out in detail the combinations of intervention categories used, the key features of cachexia targeted, and the extent of tailoring across multicomponent interventions for adults with cancer cachexia. Only a small proportion of the multicomponent interventions targeted all four key features of cachexia, but most were either partly or fully tailored to individuals. Despite sixty‐two multicomponent interventions being investigated, only four studies compared these to usual care and reported quality of life outcomes. High risk of bias, low sample sizes and variable outcome data remain challenges to the interpretability of results in this field.

## 1 Introduction

Cancer cachexia is ‘a multi‐factorial syndrome characterised by an ongoing loss of skeletal muscle mass (with or without loss of fat mass) that cannot be fully reversed by conventional nutritional support and leads to progressive functional impairment’ [1]. Key pathophysiological features of the syndrome include a negative protein and energy balance, which results from reduced food intake in combination with abnormal metabolism [1]. Cachexia is generally progressive, especially if the underlying disease cannot be cured [2]. The prevalence of cachexia amongst people with cancer varies from around 20%–80%, depending on the primary tumour site, stage and other patient factors [3, 4]. Cancer cachexia has physical, psychological, social and existential consequences for patients and carers [5]. It impacts the quality of life, with eating‐related issues and worries about physical decline contributing to negative body image, altered sense of identity, feelings of loss of control and negative emotions [6].

Although there is no current gold standard treatment for cancer cachexia, trials of interventions over the past decades have targeted different aspects of the syndrome, such as inflammation [7], appetite [8], muscle mass and function [9] and psychosocial wellbeing [10], often in isolation. The complex pathophysiology of cancer cachexia suggests the need for a multitargeted intervention. Pharmaceutical trials have developed and tested various drugs with the aim of alleviating cachexia with one single pill, some of which have shown positive effects on certain outcomes like body weight [11]. However, few drugs have shown positive effects on functional outcomes like handgrip strength or on patient‐reported outcomes like quality of life. Some trials have tested combinations of pharmacological agents, and although data on this are limited, there is some indication that these may be more effective than single drugs, highlighting the need for a multitargeted approach [11].

Therefore, the current dominant view is that a combination of interventions is required to alleviate cachexia [12, 13], a concept that was pioneered by Fearon, who proposed combining exercise, nutrition support and anti‐inflammatory drugs [14]. Since then, many trials have embraced a multimodal approach, investigating combinations of pharmacological, nutritional, exercise and/or psychosocial intervention components [15]. However, it is not known how many of these multicomponent interventions have targeted all the key features of cachexia or whether interventions that target more key features of cachexia are more effective at improving patient‐centred outcomes including quality of life. Indeed, the diversity of multicomponent interventions brings the challenge of pooling study results in meta‐analyses to evaluate which combinations of intervention components are effective at improving patient outcomes.

Furthermore, interview studies have highlighted that patients and carers value interventions that are individualised and flexible [16, 17, 18, 19, 20]. Although some trials have incorporated this approach (e.g., individualised exercise or nutritional counselling [20, 21]), usually all intervention components are provided, in one way or another, to all participants. In contrast, in nonexperimental settings like clinical services, it is more common for intervention components to only be provided if there is an indicated need (e.g., prescriptions and recommendations for present symptoms, e.g., [22]). This approach aligns with the progressive nature of cachexia; individuals with refractory cachexia will likely benefit from different intervention components than individuals with pre‐cachexia [23]. It also supports the large variability in cachexia presentation, where appetite may be the primary concern for one person, whereas muscle weakness may be the primary concern for another.

Systematic reviews of cancer cachexia interventions have often included only randomised trials with published results. Usually, nonrandomised studies of interventions (NRSIs), such as single‐arm feasibility trials and studies evaluating the effects of clinical services, and ongoing studies that have not yet published results, are excluded. However, both NRSIs and ongoing studies can contribute valuable information about the types of interventions that have been and are currently being investigated for people with cancer cachexia. In addition to this, although methodological limitations and potential biases must be considered, such as confounding [24], NRSIs with results can provide data on important patient‐centred outcomes like quality of life. If positive, results from NRSIs can encourage the design of randomised trials to test those interventions [25], so should not be overlooked.

We therefore synthesised studies of multicomponent interventions for adults with cancer cachexia. Our aims were to evaluate the extent to which multicomponent interventions have targeted the key features of cachexia and been tailored to individuals and whether this is associated with differential effects on quality of life outcomes.

## 2 Methods

### 2.1 Design

We conducted a systematic review of studies of multicomponent interventions for people with cancer cachexia. The protocol was published on PROSPERO on 25 July 2023 (CRD42023412551). We have reported this review according to the Preferred Reporting Items for Systematic Reviews and Meta‐Analysis (PRISMA) criteria.

### 2.2 Eligibility Criteria

#### 2.2.1 Population

We considered studies with evidence that (a) all participants were adults (aged ≥ 18 years) and had a diagnosis of cancer at baseline and (b) ≥ 75% of participants had or were at risk of cachexia at baseline. We applied the international consensus definition of cancer cachexia: weight loss of > 5% in the previous 6 months or BMI < 20 kg/m^2^ and ongoing weight loss of > 2% [1]. We considered participants with advanced (Stage 3 or 4) lung, upper gastrointestinal or head and neck cancers to be at risk of cachexia [3, 26, 27]. We excluded studies if any participant was < 18 years at baseline, if any participant did not have a cancer diagnosis at baseline or if there was no evidence that ≥ 75% of participants had or were at risk of cachexia at baseline based on our criteria.

#### 2.2.2 Interventions

We considered studies that investigated a multicomponent intervention, which we defined as an intervention that included at least two components (that could be individual interventions), either concurrently or sequentially. We excluded studies which investigated single‐component interventions. We did not consider anticancer treatments (e.g., chemotherapy) as intervention components; thus, if a study investigated a combination of an anticancer treatment and one additional intervention component, it was excluded. Examples of eligible multicomponent interventions included two or more separate drugs (e.g., megestrol acetate and celecoxib), one or more drugs plus a nutritional supplement (e.g., ibuprofen and fish oil), two or more nutritional interventions (e.g., fish oil and dietary counselling) or two or more nonpharmacological interventions (e.g., aerobic exercise and psychosocial support). We did not set any restrictions for comparators or outcome measures reported by studies.

#### 2.2.3 Study Design

We considered primary research studies that were randomised studies or NRSIs of interventions, in either an experimental (e.g., clinical trial) or nonexperimental setting (e.g., evaluation of a clinical service). We excluded secondary research studies (e.g., reviews) and records that did not present original data (e.g., expert opinions, letters to the editor and commentaries). We also excluded studies with fewer than five participants enrolled at baseline.

#### 2.2.4 Record Type

We considered published journal papers, pre‐prints and clinical trial register records. We considered ongoing studies without published results, if adequate information about the intervention was provided (e.g., if there was a published protocol or clinical trial register record available with a description of the intervention). We did not set any restrictions for the publication year. We considered records written in English or records written in other languages if they could be successfully translated by Google Translate.

### 2.3 Information Sources and Search Strategy

We conducted a comprehensive search strategy, which included databases, clinical trial registers and pre‐prints. We searched four databases (MEDLINE via Ovid, EMBASE via Ovid, Cochrane Central Register of Controlled Trials and CINAHL via EBSCO) from inception to 26 July 2023 and then conducted an updated search on 20 June 2024. In line with Cochrane [28] and BMJ guidance [29], we searched two clinical trial registers (ClinicalTrials.gov and World Health Organisation International Clinical Trials Registry Platform [WHO ICTRP]) on 26 July 2023 and again on 20 June 2024. We also searched MedRxiv on 26 July 2023 and again on 20 June 2024. Full search strategies can be found in Supporting Information S1.

### 2.4 Study Records

#### 2.4.1 Data Management

We imported all records from the database searches and MedRxiv into a systematic review manager (Rayyan) for screening. We imported records from clinical trial registers into an Excel spreadsheet for screening.

#### 2.4.2 Selection Process

We used Rayyan's automatic duplicate detection function to identify possible duplicate records amongst database records, and then, the primary reviewer (M.B.) manually resolved each one. The primary reviewer (M.B.) screened each record based on its title and abstract, and a second reviewer (A.M.) independently screened 10% of the records to check for consistency in decisions. The primary reviewer (M.B.) and at least one other reviewer (A.M./C.P./J.B.) independently screened each full text. In the case of conflict between the reviewers, the article was discussed between the primary reviewer (M.B.) and another independent reviewer (M.M.). To achieve consistency in the selection of articles, the final decision was ultimately made by the primary reviewer (M.B.).

#### 2.4.3 Data Collection

The primary reviewer (M.B.) extracted data from all included records, on report characteristics (e.g., publication type and year), study design, population characteristics (e.g., cancer site and stage, weight loss and cachexia status), interventions provided to each study arm, sample size and attrition. A second reviewer (C.P./A.M./R.D.) then checked all extracted data.

For each multicomponent intervention, the primary reviewer (M.B.) listed each individual component individually, alongside its intervention category (pharmacological, nutritional, exercise/physical activity or psychosocial), type (e.g., appetite stimulant, exercise and nutrition support), subtype where applicable (e.g., anamorelin, resistance training and enteral nutrition), key feature(s) of cachexia targeted and level of tailoring.

Two reviewers (M.B. and C.P./A.M./R.D.) independently extracted measures of quality of life, in addition to any stated intention to measure quality of life and outcome data (preferentially as mean and standard deviation or as reported by the study).

### 2.5 Risk of Bias in Individual Studies

Two reviewers (M.B. and C.P./A.M.) independently assessed the risk of bias for each study that provided data for the quality of life analysis. We used Cochrane tools for these assessments: For randomised trials, we used the revised tool for risk of bias in randomised trials (RoB 2), and for NRSIs, we used the Risk Of Bias in Non‐randomised Studies—of Interventions (ROBINS‐I). We were interested in the effect of assignment on the intervention (intention‐to‐treat analysis).

### 2.6 Data Synthesis and Analysis

#### 2.6.1 Summary Statistics

Firstly, we categorised studies as either randomised trials or NRSIs. Subsequently, we subcategorised NRSI study designs based on guidance from Cochrane [25] and Reeves and colleagues [30].

We calculated the median (range) sample size at baseline, the median (range) average age of participants and the median (range) average percentage weight loss of participants at baseline from all studies that reported these data.

Additionally, we calculated how many participants had lung, gastrointestinal and head and neck cancers and how many participants had Stage 3 or 4 cancer at baseline. These analyses included all participants from all studies that reported these data.

Finally, we calculated how many participants had cachexia at baseline. If the inclusion criteria of the study indicated that all enrolled participants had cachexia; then, we assumed that the number of participants with cachexia was equal to the sample size. If the study reported the number of participants with cachexia at baseline, then we used this number.

#### 2.6.2 Intervention Analysis

We used cancer cachexia guidelines from the European Society of Medical Oncology (ESMO [12]) and the American Society of Clinical Oncology (ASCO [31]), in addition to the international consensus definition of cancer cachexia [1], to define four key features of cancer cachexia for use in this review. These key features were as follows: (1) reduced energy intake (including causal factors like appetite loss, food aversion and nutrition impact symptoms), (2) altered metabolism (including systemic inflammation), (3) involuntary weight loss (including loss of lean and/or fat mass) and (4) decline in physical function (including causal factors like fatigue and loss of muscle strength).

We classified each individual intervention component into one of four ‘intervention categories’: nutritional, exercise/physical activity, pharmacological or psychosocial. We also classified each component based on which of the four key features of cancer cachexia it targeted. To illustrate intervention categories and targets, we created Sunburst diagrams using the Plotly package in Python. To visualise which intervention categories were combined and which key features of cachexia were targeted together, we created UpSet plots [32].

To assess the level of intervention tailoring, we first classified each component based on its provision; a component had ‘targeted provision’ if there was evidence that it was only given to study participants who needed it; otherwise, it had ‘total provision’. We also classified each component based on its delivery; a component was ‘individualised’ if there was evidence that it was modified for each participant; otherwise, it was ‘standardised’. Subsequently, we classified components with evidence of targeted provision and/or individualised delivery as ‘tailored’ and components with total provision and standardised delivery as ‘not tailored’. Then, if a multicomponent intervention consisted of some components that were tailored and some components that were not tailored, we classified it as ‘partly tailored’. If all the components in a multicomponent intervention were tailored, we classified it as ‘fully tailored’; if none of the components were tailored, we classified it as ‘not tailored’.

#### 2.6.3 Effects on Quality of Life

We extracted data from all studies that reported any validated measure of overall quality of life. We did not consider measures of symptom burden (e.g., FAACT anorexia–cachexia subscale) to be measures of quality of life. We developed a hierarchy to choose which measure of quality of life to include in the analysis, in the case that a study reported data for more than one measure of quality of life. The hierarchy placed composite/comprehensive measures (e.g., EORTC QLQ‐C30 overall score) higher than subscales or single‐item measures (e.g., EORTC QLQ‐C30 global health status subscale and ESAS quality of life item) and placed disease/condition‐specific measures higher than nonspecific measures.

We extracted follow‐up data from the time point which was closest to the end of the intervention. If there was no defined end to the intervention (e.g., in an ongoing clinical service), we extracted data from the final follow‐up time point. If there was a defined end to the intervention, but follow‐up data were not reported at the end of the intervention, we extracted data from the final follow‐up time point, even if this was part‐way through the intervention.

To explore the effect of multicomponent interventions on quality of life, we were interested in the mean change in quality of life score over the intervention period. If the mean change in quality of life score was reported in the study, we used these data. If the mean change in quality of life score was not reported in the study, but the mean baseline quality of life score and the mean postintervention quality of life score were reported, then we calculated the mean change in quality of life score, based on the Cochrane guidance [33]. We used a standardised version of the mean change in quality of life score (from now on referred to as the standardised mean change [SMC]) so that we could analyse data from different quality of life measures together. Additional information on the calculations we conducted can be found in Supporting Information S2.

To compare the effect of multicomponent interventions on quality of life versus usual care, we were interested in the difference in the mean change in quality of life scores (from now on referred to as the mean difference) between the study arms. If the mean difference was reported in the study, we used these data; if it was not reported in the study, we calculated it (Supporting Information S2). We used a standardised version of the mean difference (from now on referred to as standardised mean difference [SMD]) so that we could analyse data from different quality of life measures together. In line with the Cochrane guidance [33], we chose to use the adjusted Hedges *g* version of SMD. For our analysis, SPSS calculated the adjusted Hedges *g* and its 95% confidence intervals based on mean change in quality of life score and standard error.

Standard deviation is required to calculate SMCs and SMDs, but it is not always reported. If a study did not report a standard deviation, then we calculated or estimated it, where possible, using methods suggested by Cochrane [33] (Supporting Information S2).

## 3 Results

### 3.1 Results of the Search

**FIGURE 1:**
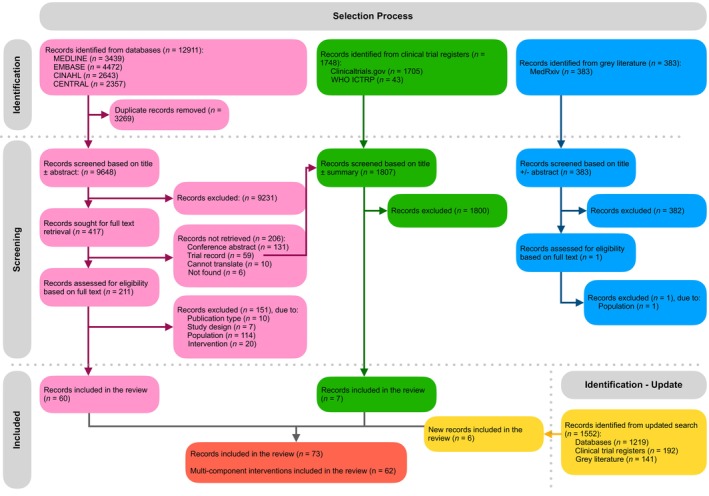
PRISMA flow diagram illustrating the identification, screening and inclusion of records in the review. Abbreviations: CENTRAL, Cochrane; WHO ICTRP, World Health Organisation International Clinical Trial Registry Platform.

The PRISMA flow diagram detailing the selection process is shown in Figure 1. In the original search, 12 911 records from databases, 1748 clinical trial register records, and 383 pre‐prints were identified. After the removal of 3269 duplicates, 11 413 records were excluded based on titles and abstracts/summaries (Figure 1). Full texts in English were successfully retrieved for 212 records. Most studies excluded at the full‐text stage were excluded because of the population; in the majority of cases, this was because there was no evidence that at least 75% of participants had or were at risk of cachexia. In the updated search, an additional 1552 records were identified and screened following the same process as the original search. Ultimately, 73 records of 62 multicomponent interventions were included in the review.

### 3.2 Included Studies

For each multicomponent intervention, we identified a main record [20, 21, 22, 34, 35, 36, 37, 38, 39, 40, 41, 42, 43, 44, 45, 46, 47, 48, 49, 50, 51, 52, 53, 54, 55, 56, 57, 58, 59, 60, 61, 62, 63, 64, 65, 66, 67, 68, 69, 70, 71, 72, 73, 74, 75, 76, 77, 78, 79, 80, 81, 82, 83, 84, 85, 86, 87, 88, 89, 90, 91, 92] alongside, in some cases, supplementary records (e.g., protocol and secondary data analysis). Tables A1 and A2 in the appendix provide overviews of the main study records for each of the 62 multicomponent interventions included in the review. The main studies were published between 1984 and 2024 and were conducted across 21 countries; 23 studies were conducted in Europe, 18 in North America, 14 in Asia, seven in Oceania and one in South America (Table A1). Of the 62 multicomponent interventions, we found published outcomes for 44 of them (Table A1); for the other 18, only a protocol or clinical trial register record was available (Table A2).

#### 3.2.1 Study Designs

Of the 62 main studies included, 39 were randomised trials and 23 were NRSIs (Tables A1 and A2). Of the NRSIs, 19 were before–after studies (uncontrolled), and four were historically controlled cohort studies. Of the 44 studies with published results, the median (range) sample size at baseline was 61.5 (6–2368) (Table A1).

#### 3.2.2 Participants

The median (range) age of participants at baseline was 64.2 (45.9–75) years (Table A1). Of the 15 studies that reported the average percentage weight loss of participants at baseline; the median (range) was 10% (2.2%–17.8%), which in most studies was over the preceding 3–6 months or compared with pre‐illness/pretreatment stable weight. Of the 4654 total participants with cancer site reported, 1591 (34%) had lung cancer, 1509 (32%) had gastrointestinal cancers, 577 (12%) had head and neck cancers and 977 (21%) had other or unspecified cancer sites (Table A1). Of the 2084 total participants with cancer stage reported, 265 (13%) had Stage 3 and 1682 (81%) had Stage 4 disease. Information on cachexia status at baseline was available for 2188 participants; of these, 1708 (78%) had cachexia or refractory cachexia (Table A1).

### 3.3 Risk of Bias in Included Studies

**FIGURE 2:**
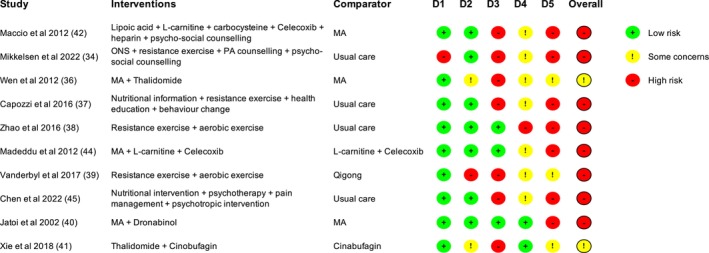
Risk of bias assessments for randomised trials, assessed using the RoB 2 tool. D1 randomisation process, D2 deviations from intended interventions, D3 missing outcome data, D4 measurement of the outcome and D5 selection of the reported result. Abbreviations: MA, megestrol acetate; ONS, oral nutritional supplements; PA, physical activity.

**FIGURE 3:**
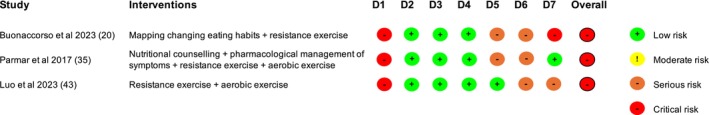
Risk of bias assessments for nonrandomised studies of interventions, assessed using the ROBINS‐I tool. D1 confounding, D2 selection of participants in the study, D3 classification of interventions, D4 deviations from intended interventions, D5 missing outcome data, D6 measurement of the outcome and D7 selection of the reported result.

The 13 studies that provided data for the quality of life analysis were assessed for risk of bias [20, 34, 35, 36, 37, 38, 39, 40, 41, 42, 43, 44, 45]. Figures 2 and 3 provide summaries of the assessments.

#### 3.3.1 Randomised Trials

We assessed eight randomised trials to be at high risk of bias overall, while the other two raised some concerns (Figure 2). Only one study had a high risk of bias relating to the randomisation process. Six studies were unblinded, in most cases, because of the nature of the intervention (e.g., exercise). Although the appropriate study population for an analysis of the intention to treat effect is all randomised participants, two studies appeared to only analyse participants who completed the trial, and in one, it was unclear who was included in the analysis.

#### 3.3.2 Nonrandomised Studies of Interventions

We assessed all three NRSIs to be at critical risk of bias overall (Figure 3). All studies had a critical risk of bias because of confounding, but all had a low risk of bias relating to the selection of participants for the study, classification of interventions and deviations from intended interventions.

#### 3.3.3 All Studies

Seven of 10 randomised trials had a high risk of bias, and two of three NRSIs had a serious risk of bias because of missing outcome data. In these studies, the ‘complete cases’ (participants for which complete data were available) may not be representative of those with missing data, potentially leading to an overestimate of the value of the intervention (inflating the within‐group quality of life change estimates). As all quality of life measures were subjective, assessment could have been influenced by knowledge of the intervention received. Therefore, given that all measures were participant‐reported, there were some concerns regarding the measurement of the outcome in all studies where participants were unblinded. In relation to the selection of the reported result, seven of 10 randomised trials had a high risk of bias, and two of three NRSIs had a critical or serious risk of bias.

### 3.4 Intervention Analysis

#### 3.4.1 Intervention Categories

From the 62 multicomponent interventions, we identified 232 individual components. Of the 232 components, 99 (43%) were nutritional, 62 (27%) were exercise/physical activity, 50 (22%) were pharmacological, and 21 (9%) were psychosocial.

**FIGURE 4:**
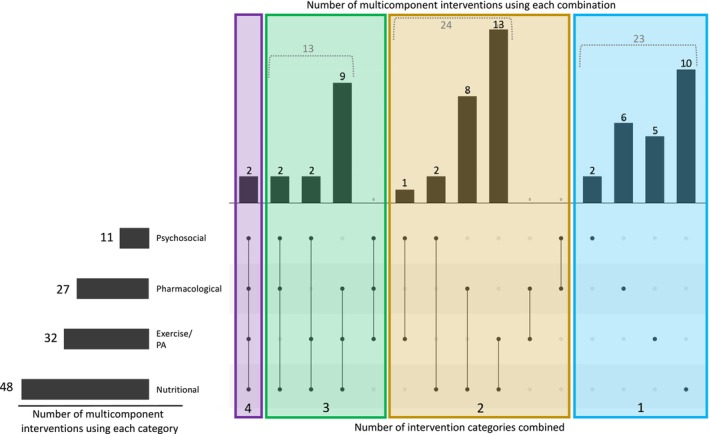
UpSet plot illustrating combinations of intervention categories used by the multicomponent interventions. The bars on the left show how many multicomponent interventions involved used each intervention category (e.g., 48 multicomponent interventions used at least one nutritional component). On the right, the matrix represents each of the possible combinations of intervention categories (from left to right: all four categories, different combinations of three categories, different combinations of two categories and each single category). The bars above the matrix show how many multicomponent interventions are employed in each of the possible combinations. Abbreviation: PA, physical activity.

Of the 62 multicomponent interventions, 48 (77%) included a nutritional component, 32 (52%) included an exercise/physical activity component, 27 (44%) included a pharmacological component and 11 (18%) included a psychosocial component (Figure 4). Two multicomponent interventions combined components from all four intervention categories [72, 76]. The most common combination of intervention categories was nutritional + exercise/physical activity, which was used in 13 multicomponent interventions.

#### 3.4.2 Key Features of Cancer Cachexia Targeted

Of the 232 components, 102 (44%) targeted energy intake, 63 (27%) targeted physical function, 64 (28%) targeted weight/muscle loss and 45 (19%) targeted metabolism.

**FIGURE 5:**
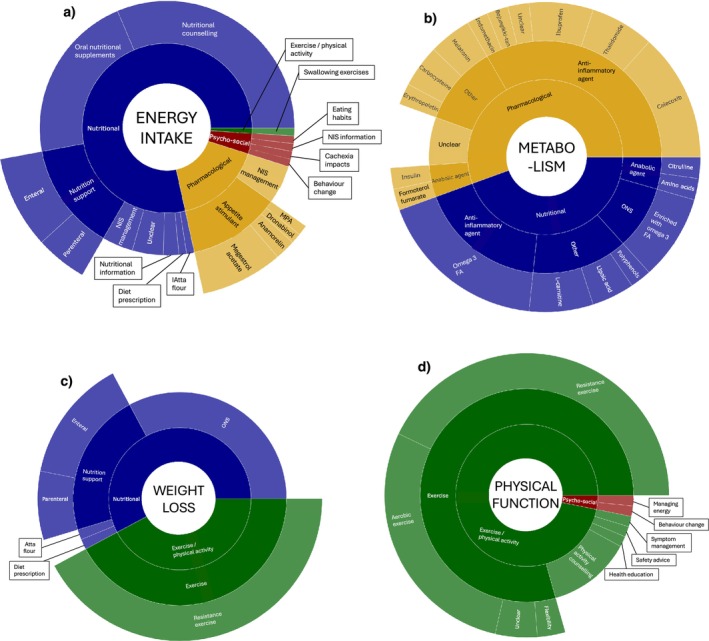
Sunburst diagrams representing the types of components that targeted (a) energy intake, (b) metabolism, (c) weight/muscle loss and (d) physical function amongst the 50 multicomponent interventions included in the review. Abbreviations: FA, fatty acid; MA, megestrol acetate; MPA, medroxyprogesterone; NIS, nutrition impact symptoms; ONS, oral nutritional supplements; PA, physical activity.

Most components targeting energy intake were nutritional (80/102, 78%), and the most common type of component was nutritional counselling (32/102, 31%; Figure 5a). Components targeting metabolism were either pharmacological (25/45, 56%) or nutritional (20/45, 44%), and over half were anti‐inflammatory agents (26/45, 58%; Figure 5b). Components targeting weight/muscle loss were either nutritional (37/64, 58%) or exercise/physical activity (27/64, 42%; Figure 5c). Most components targeting physical function were exercise/physical activity (61/63, 97%), and the most common type of component was resistance training (27/63, 43%; Figure 5d).

**FIGURE 6:**
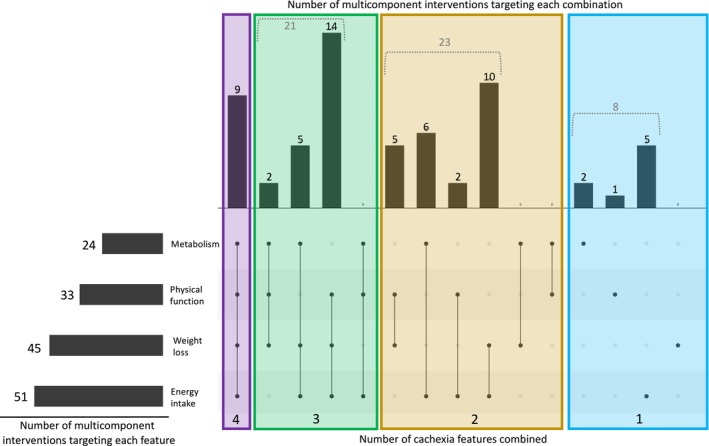
UpSet plot illustrating combinations of key features of cachexia targeted by multicomponent interventions. The bars on the left show how many multicomponent interventions targeted each key cachexia. On the right, the matrix represents each of the possible combinations of key features of cachexia targeted (from left to right: all four features, different combinations of three features, different combinations of two features and each single feature). The bars above the matrix show how many multicomponent interventions targeted each of the possible combinations of features.

Of the 62 multicomponent interventions, 51 (82%) targeted energy intake, 45 (73%) targeted weight/muscle loss, 33 (53%) targeted physical function and 24 (39%) targeted metabolism (Figure 6). Nine multicomponent interventions targeted all four key features of cachexia [22, 35, 47, 48, 49, 68, 72, 76, 83]. Fourteen multicomponent interventions targeted energy intake + weight/muscle loss + physical function, which was the most common combination of key features of cachexia targeted together.

#### 3.4.3 Tailoring

Of the 232 components, 36 (16%) had targeted provision (i.e., were only given to study participants who needed it), of which 23/36 (64%) were nutritional, 11/36 (31%) were pharmacological, and 2/36 (6%) were psychosocial. Of the 232 components, 96 (41%) were individualised (i.e., modified to each participant), of which 48/96 (50%) were exercise/physical activity, 39/96 (41%) were nutritional, and 9/96 (9%) were psychosocial. Based on these classifications, just over half of all components were tailored (126/232; 54%). Overall, 18/62 (29%) multicomponent interventions were fully tailored, 30 (48%) were partly tailored, and 14 (23%) were not tailored.

### 3.5 Effects on Quality of Life

**FIGURE 7:**
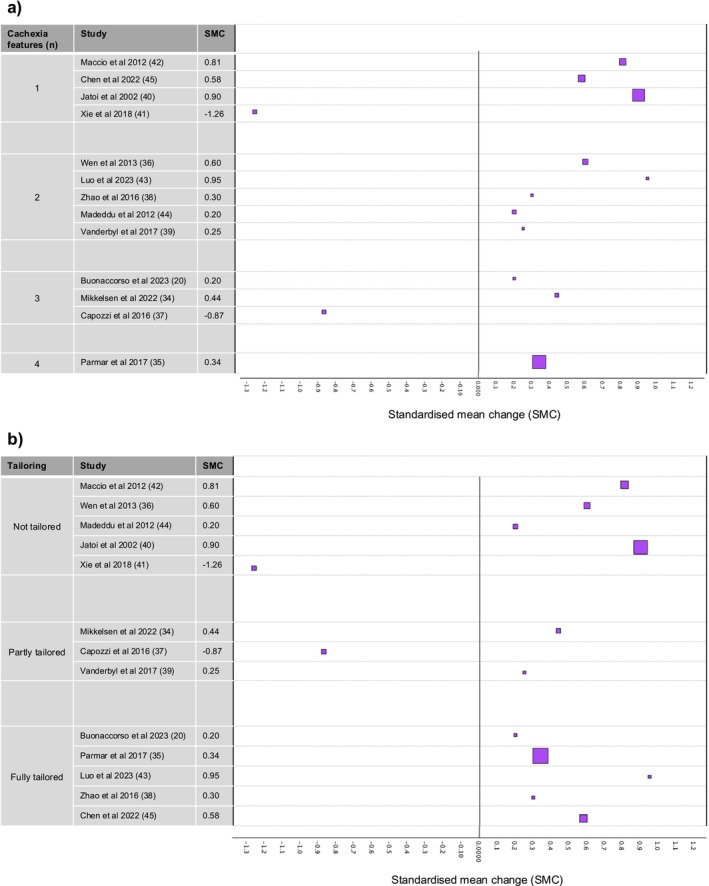
Forest plot representing standardised mean within‐arm changes in quality of life over time, for the intervention arms of 13 studies where data were available, subgrouped by (a) the number of key features of cachexia targeted by the intervention and (b) the extent to which the intervention was tailored. Larger squares indicate larger sample size. Abbreviation: SMC, standardised mean change.

Twenty‐one studies reported data on quality of life at baseline and at least one follow‐up time point and/or as change over time. However, two of these studies reported data as median and range, so we excluded them from our analysis [74, 81]. Nine studies reported within‐arm mean change in quality of life over time and its associated standard deviation (or standard error, which we converted to standard deviation) [20, 34, 35, 36, 37, 38, 39, 40, 41]. We used these data to calculate a within‐arm SMC for each intervention arm. For the remaining 10 studies, we calculated within‐arm mean change in quality of life over time as it was not reported by the study [22, 42, 43, 44, 45, 58, 61, 69, 73, 82]. However, we were only able to estimate standard deviations and therefore calculate SMCs, for four of these studies [42, 43, 44, 45]. Therefore, 13 SMCs were analysed (Figure 7).

Amongst the 13 studies, the following measures of quality of life were used: EORTC‐QLQ‐C30 standardised overall score [36, 41, 42, 44], FAACT raw overall score [20, 35, 40], FACT‐G overall score as percentage [39]; FACT‐Lung overall raw score [45], FACT‐Anaemia trial outcome index [37], MOS‐SF‐36 raw overall score [38] and EORTC‐QLQ‐C30 standardised global health status subscale score [34, 43]. For all these quality of life measures, a higher score indicated a better quality of life.

In two studies, quality of life scores declined over the intervention period [37, 41], whereas in 11 studies, quality of life scores suggested an improvement (Figure 7). SMCs varied from −1.26 to 0.95. Amongst these data, there was no indication that the number of key features of cachexia targeted, or the extent of tailoring, was associated with a greater improvement in quality of life scores.

**FIGURE 8:**
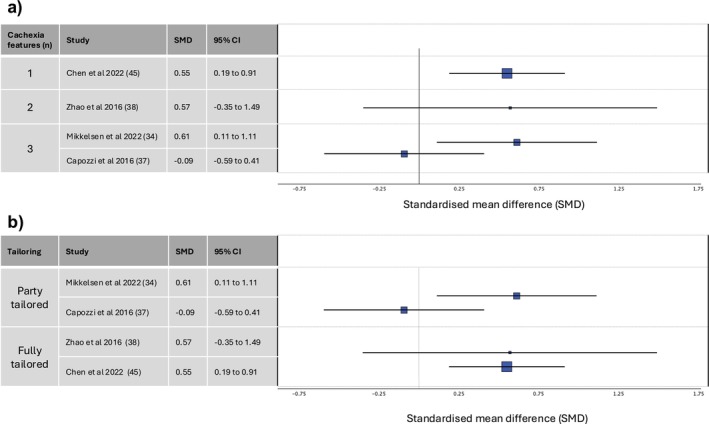
Forest plots representing standardised difference in means between study arms, for the four studies where data were available, subgrouped by (a) the number of key features of cachexia targeted by the intervention and (b) the extent to which the intervention was tailored. Larger squares indicate larger sample size. Abbreviations: CI, confidence interval; SMD, standardised mean difference.

Only four studies with quality of life data compared the multicomponent intervention with usual care [34, 37, 38, 45] (Figure 8). In three studies, the multicomponent intervention arm showed an improvement in quality of life scores, while the usual care arm showed either no change or a decline in quality of life scores [34, 38, 45]. Two of these studies reported the results of a statistical test, and in both, the difference between the two arms was statistically significant. In the fourth study, both arms showed a decline in quality of life scores, which was greater in the multicomponent intervention arm but not statistically significant [37].

## 4 Discussion

### 4.1 Summary of Key Results

We identified 62 multicomponent interventions for people with cancer cachexia. Only two interventions combined all four intervention categories (pharmacological, nutritional, exercise/physical activity and psychosocial); most included a nutritional component, whereas a minority included a psychosocial component. Nine interventions targeted all four key features of cachexia (reduced energy intake, altered metabolism, involuntary weight loss and decline in physical function); most targeted energy intake, but fewer than half targeted metabolism. We found that about half of all individual components were tailored towards individuals; in most cases, these were nutritional or exercise/physical activity components delivered in an individualised manner. Eighteen multicomponent interventions were fully tailored, and 30 were partly tailored to individuals.

Although outcomes were available for 44 interventions, only 13 reported quality of life data that could be used to calculate SMCs. In 11 of these studies, quality of life scores suggested an improvement over the intervention period, amongst participants in the multicomponent intervention arm. Four studies compared a multicomponent intervention with usual care; in three, quality of life scores suggested a greater improvement in quality of life in the intervention arm than in the usual care arm. Amongst the limited data, there was no suggestion that the number of key features of cachexia targeted, or the extent of tailoring, was associated with a greater improvement in quality of life.

### 4.2 Results in Context

To the best of our knowledge, this is the first systematic review to map out in detail the combinations of intervention categories used and key features of cachexia targeted by multicomponent interventions for adults with cancer cachexia. An intervention that combines all four intervention categories is likely to require the involvement of multiple healthcare professions, bringing challenges around organisation and funding. This may explain why one of the studies that used all four categories was a retrospective, nonexperimental study of a clinical service and the other was a small‐scale pilot study. On the other hand, more interventions targeted all four key features of cachexia. We would not have been able to determine this if only randomised trials with results had been included (which is often the case with systematic reviews) because four of the studies that targeted all four key features of cachexia were NRSIs (three nonexperimental studies of clinical services and one historically controlled cohort study) and two were protocols of randomised trials without published results. This emphasises the value of including NRSIs and ongoing studies, to increase our understanding of the types of interventions that have been and are being investigated for cancer cachexia. However, it also highlights the challenges of evaluating the effectiveness of these interventions.

As nutritional aspects of cachexia have dominated the literature and interventions have generally taken a biomedical approach, we were not surprised that a high proportion of multicomponent interventions involved a nutritional component and/or targeted energy intake. However, we had expected a higher proportion of the interventions to target metabolism. Meanwhile, only 11 multicomponent interventions involved a psychosocial component, which is likely because the psychosocial impacts of the syndrome have only become a focus of cachexia literature more recently. Interview studies exploring experiences of cancer cachexia have repeatedly reported themes of eating‐related distress and family conflicts, in part because of a lack of understanding about cachexia and the role of food in reversing weight loss [5, 6]. Participants receiving psychosocial/educational interventions have reported improved health literacy, awareness about nutrition and relationships with food in recent interview studies [19, 20]. These positive experiences suggest that some of the key quality of life issues around eating may be effectively tackled with psychosocial interventions rather than, or in addition to, nutritional interventions. Therefore, we believe that future interventions for people with cancer cachexia should include a psychosocial component. Going forward, the recently developed patient‐reported outcome measure to assess eating‐related distress [103] could be used in addition to qualitative data from interview studies, to evaluate the effects of interventions on this key quality of life issue amongst people with cancer cachexia.

We believe this is also the first review to systematically assess tailoring across interventions for people with cancer cachexia. Over a quarter of the multicomponent interventions were fully tailored, and almost half were partly tailored. This, in part, was due to the inclusion of NRSIs (10 out of the 18 fully tailored interventions and 11 out of the 30 partly tailored interventions were NRSIs), particularly nonexperimental studies of clinical services, which often provided intervention components based on indicated need. Interview studies have found that patients want and value interventions that are individualised and flexible [16, 19, 20], so we believe that future interventions should take a tailored approach. Ideally, this should not be limited to NRSIs and be incorporated into large‐scale clinical trials, so that the effectiveness of tailored interventions can be evaluated with sufficient power to detect effects and without the methodological limitations and potential biases of NRSIs.

We had expected more of the 62 multicomponent interventions to report data on quality of life and were surprised at the number of studies that did not intend to measure it. Assessment of quality of life in interventional studies is an important part of conducting patient‐centred research and whether patients perceive a benefit should be a key consideration towards whether an intervention is deemed to be successful or not [104]. It is a particularly relevant outcome for the cancer cachexia population, who often have incurable cancers and/or irreversible cachexia and so outcomes like survival are not appropriate measures of intervention success. Furthermore, this population experiences multidimensional quality of life issues related to both the nutritional and functional aspects of cachexia [104, 105]. Therefore, measures of overall quality of life that encompass these domains should be reported in addition to subscales and specific symptoms that are relevant to the population, like eating‐related distress.

We were encouraged by the fact that 11 out of the 13 studies that we could calculate SMCs for reported that participants in the multicomponent intervention arm experienced an improvement in quality of life over the intervention period. However, we believe that this result comes with a high risk of bias and should be interpreted with caution. There were many studies that measured quality of life but did not report the data (or only reported subscales and not the overall score) and so could not be included in our analysis. Additionally, we assessed 11 out of the 13 studies in our analysis as having a high risk of bias. Much of this risk of bias is difficult to avoid in this field, in part because of the nature of the interventions (blinding is often not possible), the nature of the outcome measure (quality of life being a subjective and participant‐reported measure, where participants are usually aware of the intervention received, leading to risk of bias in measurement of the outcome), and the population (high levels of attrition due to worsening health status and death, leading to risk of bias due to missing outcome data). However, nine out of the 13 studies had a high risk of bias because of the selection of the reported result, which can be controlled by the investigators. In addition, there may be publication bias whereby studies showing improvements in outcomes like quality of life were more likely to be published than studies not showing improvements. As a result, we believe that (a) the SMCs in our analysis are likely to be overestimates and (b) the number of studies of multicomponent interventions in which participants experienced a decline in quality of life over the intervention period is likely to be higher than is shown in this review.

Furthermore, although there was no indication that the number of key features of cachexia targeted, or the extent of tailoring, was associated with a greater improvement in quality of life scores amongst these data, the heterogeneity across the 13 studies prevented us from concluding on our hypothesis. Therefore, to better understand the effects of multicomponent interventions on quality of life for people with cancer cachexia, we need more robust and rigorous randomised trials that measure quality of life and report it appropriately and transparently.

Although this systematic review was being conducted, a new Phase 2 drug trial testing the efficacy and safety of ponsegromab was published [106]. As pharmaceutical trials such as this one continue their efforts to develop a drug that effectively alleviates cachexia, we believe that nonpharmacological interventions remain an important focus of cachexia research. Ponsegromab has shown promising early results, with participants gaining weight over 12 weeks and reporting reduced symptom burden. However, the inclusion criteria for participants were strict (including GDF‐15 levels of at least 1500 pg/mL), and the list of exclusion criteria was long, which led to over a quarter of the participants initially enrolled on the trial being excluded. This highlights the need for a range of interventions to be available, so that suitable options exist for everybody with cachexia.

### 4.3 Strengths and Limitations of the Review Methods

As there is no agreed framework for the key features of cancer cachexia, we devised four key features for use in this review. We identified these key features based on the consensus definition of cancer cachexia [1] and guidelines from ASCO [31] and ESMO [12], which ensured that they were strongly aligned with current understanding and agreement about what cachexia is. However, the psychosocial impact of cachexia on patients is not represented in these definitions, so we did not include it as a key feature of cachexia in this review. Instead, ‘psychosocial’ was included as an intervention category. However, targeting the psychosocial impact of cachexia in addition to the four key features could lead to greater improvements in quality of life, as indicated by qualitative findings of interventional studies [19, 20]. As it was not assessed in this review, future work could explore whether interventions targeting the psychosocial impacts of cachexia have greater effects on quality of life than interventions which do not.

We classified each intervention component based on which key feature(s) of cachexia it *targeted* and not on which feature(s) it *impacted*. For example, we classified aerobic exercise as *targeting* physical function, but we acknowledge that it is also likely to *impact* the other three features (metabolism, energy intake and weight/body composition). This distinction was necessary for the purposes of the review, to avoid classifying all interventions as having an impact on all four features. However, biological connections between the features of cachexia are important to consider, to fully understand which interventions may be effective at improving this multifactorial syndrome.

We included studies where there was evidence that the population either had cachexia or was at risk of cachexia. We took this approach because if the review had been restricted only to studies that assessed potentially eligible participants for cachexia, we would have excluded many relevant studies. We used cancer stage and site as indicators of cachexia risk. Published data show that certain cancers have a much higher prevalence of cachexia than others [3, 27]. Furthermore, as cachexia is a progressive syndrome, it is generally agreed that cachexia increases with cancer severity [107, 108, 109]. Although there are some data supporting this [111, 112], we acknowledge that this is not universal and that cachexia can present in earlier stages of disease. However, no indicator of cachexia risk is perfect, and other clinical characteristics, such as nutritional status or chronic comorbidities, may also indicate the risk of cachexia.

Furthermore, we did not exclude studies based on the intended target of the intervention. For example, if a study met all our eligibility criteria and stated that their multicomponent intervention was aimed at improving fatigue or poor appetite, we included it in the review. We made this decision because the aim of this review was to evaluate multicomponent interventions that have been tested amongst people with (or at risk of) cancer cachexia, rather than only evaluating multicomponent interventions explicitly aimed at cachexia. However, this meant that some studies included in the review investigated interventions that were not necessarily designed with cachexia in mind. This could be one of the reasons that some interventions targeted only one or two of the key features of cachexia, so should be considered when interpreting the results of the review.

We used standardised means to represent change over time and difference between arms (SMCs and SMDs). This approach allowed us to compare the effect on quality of life across studies that used different measurement instruments, which was necessary to explore differential effects on quality of life. However, this approach also introduced some limitations. First, it meant excluding eight studies that reported data on quality of life but for which SMCs could not be calculated. Second, there were small sample sizes in many studies, which can lead to higher standard deviations and therefore lower SMCs. When compared to larger studies with smaller standard deviations, this may skew the results in favour of the larger studies.

### 4.4 Summary of Recommendations

We believe that future interventions for people with cancer cachexia should include a psychosocial component and should take a tailored approach. Although challenging, these approaches should not be limited to NRSIs and small pilot studies, as we need large‐scale, robust randomised trials to properly evaluate the effects of multicomponent interventions. Furthermore, studies should measure overall quality of life in addition to quality of life subscales and specific measures relating to key quality of life issues and symptoms experienced by this population, such as eating‐related distress. These measures need to be reported at multiple time points, appropriately and transparently, to reduce the risk of bias and allow for secondary data analyses to be conducted, such as meta‐analyses.

## 5 Authors' Conclusions

This review mapped out in detail the combinations of intervention categories used, key features of cachexia targeted and the extent of tailoring across multicomponent interventions for adults with cancer cachexia. Only nine of the 62 multicomponent interventions targeted all four key features of cachexia, but 48 were either partly or fully tailored to individuals. Only four studies compared a multicomponent intervention to usual care and reported quality of life outcomes ‐ while three of these showed a positive effect of the intervention, high risk of bias, low sample sizes and variable outcome data remain challenges to the interpretability of these results.

## Ethics Statement

The authors of this manuscript certify that they comply with the ethical guidelines for authorship and publishing in the Journal of Cachexia, Sarcopenia and Muscle [112]. The manuscript does not contain clinical studies or patient data.

## Conflicts of Interest

The authors declare no conflicts of interest.

## Differences Between Protocol and Review

During the screening process, it became apparent that more specific population eligibility criteria were needed to ensure reproducibility and consistency regarding which studies were included in the review and which were excluded. Therefore, we decided to use a numerical cut‐off, whereby studies with at least 75% of participants at baseline with evidence of cachexia or risk of cachexia were included. In the registered protocol, we stated that only comparative studies would be included in the analysis of clinical effectiveness. However, because of the limited number of studies for which quality of life data were available for an intervention and control arm, we decided to expand our analysis. We therefore analysed the effect of interventions on quality of life over time within study arms, to allow for the inclusion of single‐arm studies, in addition to analysing comparative studies as planned. We had planned to analyse five outcome domains: one primary (quality of life) and four secondary (energy intake, weight/body composition, metabolism and physical function). However, upon data extraction of these outcomes, it became clear that it would be too much to investigate all five of them in this systematic review. Therefore, we decided to focus only on the primary outcome of quality of life in this review.

## Supporting information


**Table S1.1** Search strategy for MEDLINE via Ovid.
**Table S1.2** Search strategy for EMBASE via Ovid.
**Table S1.3** Search strategy for CINAHL via EBSCO.
**Table S1.4** Search strategy for Cochrane Central Register of Controlled Trials.
**Table S1.5** Search strategy for ClinicalTrials.gov.
**Table S1.6** Search strategy for WHO ICTRP.
**Table S1.7** Search strategy for MedRxiv.

**Data S1** Supporting Information.

## Appendix A

**TABLE A1:** Characteristics of included multicomponent interventions (with published results).

Main record	Supplementary records	Study design (*n* of arms)	Country (*n* of centres)	*n*	Average age*	Males, *n* (%)	Primary cancer sites, *n*	Cancer stages, *n*	Cachexia or weight loss screening/classification criteria	% Weight change (time)	Cachexia prevalence, *n*	Intervention components	Comparator
Naito et al. [21]	Mouri et al. [93]	NRSI—before–after study (1 arm)	Japan (4 centres)	30	75 (70–85)	20 (67%)	Lung *n* = 24, pancreatic *n* = 6	III *n* = 3 and IV or postoperative recurrence *n* = 27	Unintentional WL > 5% in last 6 months or WL > 2% and BMI < 20 or presence of muscle depletion according to consensus criteria	−3.0 ± 6.8 (6 months)	Cachexia *n* = 12	Nutritional counselling + ONS + resistance exercise + PA counselling	None (uncontrolled)
Solheim et al. [48]	Balstad et al. [94]	Randomised trial (2 arms)	Norway and United Kingdom (3 centres)	46	Intervention: 63.0 (54.5–68.0) and control: 59.0 (52.5–67.0)^d^	26 (57%)	Lung *n* = 26, pancreatic *n* = 20	III *n* = 16 and IV *n* = 30	BMI < 30 kg/m^2^ and < 20% WL in last 6 months	Intervention arm: −5.7 and control arm: −5.4 (6 months)^a^	Cachexia *n* = 46	Nutritional counselling + ONS + omega 3 FA + celecoxib + resistance exercise + aerobic exercise	Standard care
Maccio et al. [42]	NA	Randomised trial (2 arms)	Italy (4 centres)	144	60.5 ± 12.74	Not specified	Ovary *n* = 50, endometrium *n* = 49 and cervix *n* = 25	IIIC *n* = 5 and IV *n* = 119	Loss of at least 5% of ideal or pre‐illness BW in the last 3 months	< −5% *n* = 2, −5% to −10% *n* = 69, > −10 *n* = 53 (3–6 months)	Cachexia *n* = 144	Lipoic acid + L‐carnitine + carbocysteine + Celecoxib + heparin + psychosocial counselling	MA + heparin + psychosocial counselling
Buonaccorso et al. [20]	Buonaccorso et al. [95]	NRSI—before–after study (1 arm)	Italy (1 centre)	24	65.96 ± 10.61	15 (63%)	Pancreatic *n* = 6, lung *n* = 5, renal *n* = 3, upper GI *n* = 3, bladder *n* = 2 and other *n* = 5	Not specified	Refractory cachexia and cachexia based on ESPEN guidelines and MUST	Not specified	Reversible cachexia *n* = 20 and refractory cachexia *n* = 4	Mapping changing eating habits + resistance exercise	None (uncontrolled)
Mikkelsen et al. [34]	Mikkelsen et al. [96]	Randomised trial (2 arms)	Denmark (1 centre)	84	Intervention 72.1 (67.3–74.5), control arm 71.5 (68.5–75.3)^d^	36 (43%)	Lung *n* = 39, pancreatic *n* = 36 and biliary tract *n* = 9	Locally advanced *n* = 12 and metastatic *n* = 72	None specified	Not specified	Unknown	ONS + resistance exercise + PA counselling + psychosocial counselling	Standard oncological treatment
Bland et al. [22]	NA	NRSI—before–after study—retrospective (1 arm)	Australia (1 centre)	162	67.2 ± 12.0	94 (58%)	Upper GI *n* = 49, lung *n* = 38, colorectal *n* = 24, prostate *n* = 17, H&N *n* = 8 and other *n* = 26	Metastatic *n* = 120 and locally advanced *n* = 42	Pre‐cachexia: WL > 1 kg but < 5%. Cachexia: WL > 5% or WL > 2% and BMI < 20 kg/m^2^. Refractory cachexia: WL > 15% and BMI < 23 kg/m^2^ or WL > 20% and BMI < 27 kg/m^2^	−10.4 ± 9.4 (6 months)	Pre‐cachexia *n* = 7, cachexia *n* = 83 and refractory cachexia *n* = 29	Nutritional counselling + ONS + pharmacological management of NIS, malabsorption and inflammation if indicated + resistance exercise + PA counselling + fatigue/breathlessness management + safety advice	None (uncontrolled)
Chang [50]	NA	NRSI—before–after study—retrospective (1 arm)	Taiwan (1 centre)	126	Under 65 group: 48 [3, 26, 27, 28, 29, 30, 31, 32, 33, 34, 35, 36, 37, 38, 39, 40, 41, 42, 43, 44, 45, 46, 47, 48, 49, 50, 51, 52, 53, 54, 55, 56, 57, 58, 59, 60, 61, 62, 63], 65 + group 68 [64, 65, 66, 67, 68, 69, 70, 71, 72, 73, 74, 75]	116 (92%)	H&N *n* = 126	III *n* = 24 and IV *n* = 102	None specified	Not specified	Not specified	EN + NIS management + vitamins	None (uncontrolled)
Greig et al. [51]	NA	NRSI—before–after study (1 arm)	United Kingdom (1 centre)	13	65.46 ± 9.84	5 (38%)	Pancreatic *n* = 5, gastric *n* = 2, cholangiocarcinoma *n* = 2, oesophageal *n* = 2, rectal *n* = 1 and mesothelioma *n* = 1	IV *n* = 13	WL ≥ 2% in 2 months or ≥ 5% in 6 months	−10.95 ± 4.42 (mean of 4.72 months)	Cachexia *n* = 13	MA + formoterol fumarate	None (uncontrolled)
Parmar et al. [35]	Chasen et al. [97], Gagnon et al. [98], Parmar et al. [99], Eades et al. [100]	NRSI—before–after study—retrospective (1 arm)	Canada (1 centre)	405	65.2 ± 13.3	208 (51%)	Lung *n* = 137, GI *n* = 104, haematological *n* = 48, breast *n* = 24 and miscellaneous *n* = 61	Not specified	WL, anorexia or generalised functional decline; cachexia classification of participants based on international consensus definition	−10.2 ± 9.8 (6 months)	Pre‐cachexia *n* = 64, cachexia *n* = 257	Nutritional counselling + pharmacological management of symptoms if indicated + resistance exercise + aerobic exercise	None (uncontrolled)
Del Fabbro et al. [52]	NA	NRSI—before–after study—retrospective (1 arm)	United States (1 centre)	151	60 (19–86)	95 (63%)	GI *n* = 58, respiratory *n* = 33, genitourinary *n* = 16, H&N *n* = 15 and other *n* = 29	IV *n* = 147	History of WL ≥ 5% and/or complained of poor appetite	−9 (100 days)^a^	Cachexia *n* = 151	Nutritional counselling + exercise recommendations	None (uncontrolled)
Wen et al. [36]	NA	Randomised trial (2 arms)	China (1 centre)	108	61.95^c^	55 (51%)	Lung *n* = 34, gastric *n* = 13, breast *n* = 13, colorectal *n* = 11, liver *n* = 8, pancreas *n* = 7 and oesophagus *n* = 7	III *n* = 10 and IV *n* = 83	Loss of > 5% of pre‐illness or ideal BW in the last 3 months	−5% to −10% *n* = 75	Cachexia *n* = 108	MA + thalidomide	MA
Bibby et al. [53]	NA	NRSI—before–after study—retrospective (1 arm)	United Kingdom (1 centre)	137	69 (35–86)	70 (51%)	Pancreas *n* = 137	Not specified	None specified	−6.3 (−27.7 to +10.4) (time unspecified)	Unknown	Nutritional counselling + ONS + EN or PN if indicated + PA counselling	None (uncontrolled)
Kufeldt et al. [54]	NA	NRSI—historically controlled cohort study (2 arms)	Germany (1 centre)	2368	62.99 ± 12.31	555 (23%)	Bronchial, breast, prostate, oesophageal, cervical, ENT, lymphoma, glioblastoma and other (*n* not specified)	Not specified	None specified	Up to −39.2% *n* = 569 (of *n* = 666)	Unknown	Nutritional counselling + EN or PN if indicated	Historical control
Hajdu et al. [56]	NA	Randomised trial (2 arms)	Denmark (2 centres)	69	62 (39–80)^b^	57 (83%)	H&N *n* = 69	I *n* = 9, II *n* = 7, III *n* = 10 and IV *n* = 43	None specified	Not specified	Unknown	Swallowing exercises + ONS + resistance exercise	Standard cancer care
Kouchaki et al. [58]	NA	Randomised trial (2 arms)	Iran (1 centre)	90	60.92^c^	56 (62%)	Gastric *n* = 47, colorectal *n* = 26, oesophageal *n* = 14 and pancreatic *n* = 3	Metastasis *n* = 30 and no metastasis *n* = 60	At least 5% unintentional WL in the last 6 months or relative to premorbid stable weight or BMI < 20	−17.45 (relative to premorbid historical stable weight)^c^	Cachexia *n* = 90	MA + Celecoxib	MA + placebo
Percival et al. [59]	NA	NRSI—before–after study (1 arm)	United Kingdom (1 centre)	243	70 ± 10	139 (57%)	Lung *n* = 219, clinical diagnosis *n* = 14 and mesothelioma *n* = 10 (all thoracic)	I *n* = 30, II *n* = 26, III *n* = 52, IV *n* = 93. local *n* = 8 and extensive *n* = 34	None specified	5 (a). > −10% *n* = 59	Cachexia *n* = 59	Nutritional counselling + ONS	None (uncontrolled)
Sykes et al. [60]	NA	Randomised trial (2 arms)	United States (1 centre)	55	63.2 ± 11.8	30 (55%)	H&N *n* = 55	I or II *n* = 5 and III or IV *n* = 43	None specified	Not specified	Unknown	Nutritional counselling + nutritional information + ONS + omega 3 FA	Standard care
Capozzi et al. [37]	NA	Randomised trial (2 arms)	Canada (1 centre)	60	56.1 ± 9.2	49 (81.7%)	H&N *n* = 60	I *n* = 1, II *n* = 7, III *n* = 3 and IV *n* = 48	None specified	Not specified	Unknown	Nutritional information + resistance exercise + health education + behaviour change	Waitlist control
Daly et al. [62]	NA	Randomised trial (2 arms)	United States (2 centres)	40	54^c^	32 (80%)	H&N *n* = 40	II or nonstaged recurrence *n* = 3, III *n* = 9 and IV *n* = 28	None specified	−7.15 (time unspecified)^c^	Unknown	Nutritional counselling + ONS if indicated + pharmacological management of NIS if indicated	EN + nutritional counselling
Orell et al. [63]	NA	Randomised trial (2 arms)	Finland (1 centre)	65	Intervention 57 (52–64), control 61 (56–64)^d^	46 (79%)	H&N *n* = 65	I *n* = 2, II *n* = 6, III *n* = 12, IV *n* = 37 and unknown *n* = 1	None specified	Not specified	Unknown	Nutritional counselling + EN	On‐demand nutritional counselling
Luo et al. [43]	NA	NRSI—before–after study (1 arm)	Australia (1 centre)	6	68.17 ± 6.05	5 (83%)	Pancreas *n* = 6	Metastatic *n* = 4 and locally advanced *n* = 2	None specified	Not specified	Unknown	Resistance exercise + aerobic exercise	None (uncontrolled)
Zhao et al. [38]	NA	Randomised trial (2 arms)	United States (2 centres)	20	57^c^	17 (94%)	H&N *n* = 20	III *n* = 4 and IV *n* = 14	None specified	Not specified	Unknown	Resistance exercise + aerobic exercise	Standard treatment
Yennurajalingam et al. [64]	NA	NRSI—before–after study (1 arm)	United States (1 centre)	45	64^a^	18 (64.3%)	Lung *n* = 17, bile duct *n* = 2, colon *n* = 2, pancreas *n* = 2, breast *n* = 2, gynaecological *n* = 2 and H&N *n* = 1	Advanced *n* = 45	Unintentional WL from ≥ 2% to ≤ 15% at any time in the last 12 months	Not specified	Unknown	Nutritional counselling + ONS + anamorelin + zinc + resistance exercise + aerobic exercise	None (uncontrolled)
Lundholm et al. [65]	NA	Randomised trial (2 arms)	Sweden (1 centre)	138	70^c^	Not specified	Oesophageal/gastric *n* = 53, pancreatic *n* = 44, colorectal *n* = 15, liver/bile duct *n* = 12, miscellaneous *n* = 9 and unknown *n* = 5	Not specified	Manifest WL due to generalised malignant disease	−10 (1)^e^	Unknown	Nutritional counselling + ONS if indicated + PN if indicated + insulin if indicated + indomethacin + erythropoietin	Best available palliative support
Persson et al. [67]	NA	Randomised trial (2 arms)	Sweden (1 centre)	24	67.5^c^	10 (42%)	Pancreatic *n* = 8, colorectal *n* = 8, biliary *n* = 4, gastric *n* = 2 and adenocarcinoma or unknown primary origin *n* = 2 (all GI)	Not specified	> 10% WL in last 6 months	−12 (6 months)^c^	Cachexia *n* = 24	Nutritional counselling + melatonin + omega 3 FA	Omega 3 FA only *OR* melatonin only
Tobberup et al. [68]	NA	NRSI—historically controlled cohort study (2 arms)	Denmark (1 centre)	120	67.75^c^	71 (59%)	Lung *n* = 120	I–IIb *n* = 5, IIIa–IIIb *n* = 31 and IV *n* = 84	Pre‐cachexia: any WL < 5% and presence of anorexia; cachexia: WL > 5% in last 6 months or ongoing WL > 2% and BMI < 20 or low skeletal muscle mass, < 55 cm^2^/m^2^ for men and < 39 cm^2^/m^2^ for women	−2.2 (compared to pretreatment weight)^c^	Cachexia *n* = 40, pre‐cachexia *n* = 14, no cachexia *n* = 66	Nutritional counselling + omega 3 FA + resistance exercise + aerobic exercise	Historical control
Matsumoto et al. [69]	NA	NRSI—before–after study (1 arm)	Japan (1 centre)	50	66 (40–78)	42 (84%)	Lung *n* = 50	IV or extensive disease *n* = 50	None specified	Not specified	Unknown	Nutritionist referral if indicated + information + physical, psychological or socio‐economic intervention if indicated	None (uncontrolled)
Glare et al. [71]	NA	NRSI—before–after study (1 arm)	Australia (1 centre)	54	62 (24–85)	36 (68%)	Lung *n* = 18, colorectal *n* = 9, upper GI (oesophageal and gastric) *n* = 9, hepatopancreatobiliary *n* = 8, breast *n* = 4, prostate *n* = 2 and other *n* = 4	Not specified	None specified	−10.2 (0–27) (6 months)	Unknown	Nutritional intervention + resistance exercise + aerobic exercise	None (uncontrolled)
Vaughan et al. [72]	NA	NRSI—before–after study—retrospective (1 arm)	Australia (1 centre)	175	71 (33–91)	107 (61%)	Lung *n* = 57, colorectal *n* = 17, pancreas *n* = 13, H&N *n* = 11, other GI *n* = 22, prostate *n* = 13, breast *n* = 9, other urogenital *n* = 10 and other *n* = 23	I *n* = 2, II *n* = 3, III and loco‐regional disease not resectable *n* = 15, IV *n* = 130 and advanced disease stage not determined *n* = 25	Concern of WL	Not specified	Unknown	Nutritional counselling + ONS + nutritional management of NIS if indicated (e.g., zinc) + education on cachexia impacts + omega 3 FA if indicated + pharmacological management of symptoms if indicated + exercise + vitamins	None (uncontrolled)
Mantovani et al. [73]	Mantovani et al. [101]	Randomised trial (5 arms)	Italy (not specified)	332	61.94^c^	181 (55%)	Lung *n* = 72, breast *n* = 55, colorectum *n* = 45, pancreas *n* = 32, H&N *n* = 28, ovary *n* = 26, stomach *n* = 17, uterus *n* = 10, kidney *n* = 10, biliary ducts *n* = 8, bladder *n* = 7, prostate *n* = 7, oesophagus *n* = 8 and liver *n* = 7	III *n* = 16 and IV *n* = 317	Loss of > 5% of ideal or pre‐illness BW in last 3 months with or without abnormal values of pro‐inflammatory cytokines predictive of onset of clinical cachexia	< −5%: *n* = 76, −5 to −10%: *n* = 188 and > −10%: *n* = 68 (3–6 months)	Cachexia *n* = 332	MA/MPA + thalidomide + polyphenols + lipoic acid + carbocysteine + omega 3 FA + L‐carnitine + vitamins	Polyphenols + lipoic acid + carbocysteine + vitamins + one of MA/MPA *OR* omega 3 FA *OR* L‐carnitine *OR* thalidomide
Jatoi et al. [74]	NA	Randomised trial (3 arms)	United States and Canada (26 centres)	421	65.67^c^	293 (70%)	Lung *n* = 166, GI *n* = 141 and other *n* = 114	Not specified	Self‐reported, 2‐month WL ≥ 5 lb (2.3 kg) and/or physician‐estimated caloric intake of < 20 cal/kg of BW per day	Not specified	Unknown	ONS + MA + omega 3 FA	MA + placebo *OR* omega 3 FA + placebo
Madeddu et al. [44]	NA	Randomised trial (2 arms)	Italy (1 centre)	60	64.45^c^	33 (55%)	H&N *n* = 13, lung *n* = 12, colorectal *n* = 7, stomach *n* = 7, ovary *n* = 7, pancreas *n* = 6, oesophagus *n* = 2, biliary ducts *n* = 1 and liver *n* = 1	IIIB *n* = 3 and IV *n* = 53	Loss of ≥5% of ideal or pre‐illness BW in last 6 months (associated with abnormal values of pro‐inflammatory cytokines and/or CRP protein)	< −5%: *n* = 3, −5% to −10%: *n* = 29 and > −10%: *n* = 24 (time unspecified)	Cachexia *n* = 60	MA + L‐carnitine + celecoxib	L‐carnitine + celecoxib
Odelli et al. [75]	NA	NRSI—historically controlled cohort study (2 arms)	Australia (1 centre)	48	69.15^c^	33 (69%)	Oesophagus *n* = 48	Not specified	None specified	−7.35 (compared with usual weight)^c^	Unknown	Nutritional counselling + EN if indicated	Historical control
Ester et al. [61]	NA	NRSI—before–after study (1 arm)	Canada (1 centre)	10	64.4 ± 10.3	4 (40%)	Lung *n* = 10	IV *n* = 10	None specified	Not specified	Unknown	Nutritional counselling + health behaviour change + resistance exercise + aerobic exercise + flexibility exercise	None (uncontrolled)
Cerchietti et al. [76]	NA	NRSI—before–after study (1 arm)	Argentina (1 centre)	15	58.8 (40–76)^b^	11 (73%)	Lung *n* = 15	IIIb *n* = 2 and IV *n* = 13	Presence of cancer cachexia (i.e., ≥ 10% WL plus anorexia), fatigue, WHO PS ≥ 2 and evidence of acute‐phase response assessed by a level of CRP > 10 μg/mL	−17.8 (10–36.5) (compared to historical stable weight)	Cachexia *n* = 15	MA + EN + celecoxib + exercise + psychological/psychiatric intervention if indicated	None (uncontrolled)
Wang et al. [77]	NA	NRSI—historically controlled cohort study (2 arms)	Taiwan (1 centre)	58	53.5^c^	54 (93%)	H&N *n* = 58	IVA *n* = 37 and IVB *n* = 21	None specified	Not specified	Unknown	Nutritional counselling + ONS + EN	No nutrition support (historical control)
Vanderbyl et al. [39]	NA	Randomised trial—crossover (2 arms)	Canada (1 centre)	36	64.9^c^	14 (38%)	Lung *n* = 12 and GI *n* = 12	III *n* = 8 and IV *n* = 16	None specified	Not specified	Unknown	Resistance exercise + aerobic exercise	Qigong
Chen et al. [45]	NA	Randomised trial (2 arms)	China (1 centre)	120	63.02^c^	81 (68%)	Lung *n* = 120	IIIB *n* = 14, IIIC *n* = 18 and IV *n* = 88	None specified	Not specified	Unknown	Nutritional intervention as indicated + psychotherapy + pain management if indicated + psychotropic intervention if indicated	Standard care
Jatoi et al. [40]	NA	Randomised trial (3 arms)	United States (20 centres)	485	66.33^c^	308 (66%)	Lung *n* = 208, GI *n* = 139 and other *n* = 122	Not specified	Self‐reported WL ≥ 5 pounds (2.3 kg) in the last 2 months and/or physician‐estimated caloric intake of < 20 cal/kg of BW per day	Not specified	Unknown	MA + dronabinol	MA only *OR* dronabinol only
Xie et al. [41]	NA	Randomised trial (2 arms)	China (1 centre)	54	56.84 (46–66)^b^	42 (75%)	Lung *n* = 54	IV *n* = 54	WL > 5% or BMI < 20 kg/m^2^ in 6 months and WL > 2% or appendicular SMI matched with sarcopenia (male < 7.26 kg/m^2^, female < 5.45 kg/m^2^) having lost > 2% weight	Not specified	Cachexia *n* = 54	Thalidomide + cinobufagin	Cinobufagin
Bakitas et al. [79]	Bakitas et al. [102]	Randomised trial (2 arms)	United States (1 centre)	332	65.3^c^	168 (60%)	GI tract *n* = 119, lung = 93, genitourinary tract *n* = 37 and breast *n* = 30	Advanced *n* = 332	None	Not specified	Unknown	Education on cancer + problem‐solving + communication and social support + symptom management + advance care planning	Care as usual
Kapoor et al. [81]	NA	Randomised trial (2 arms)	India (1 centre)	63	45.9^c^	0 (0%)	Anorectum *n* = 5, bone *n* = 3, brain *n* = 1, breast *n* = 14, buccal cavity *n* = 2, chest wall *n* = 2, eyelid *n* = 1, genitourinary tract *n* = 22, lung *n* = 6, olfactory *n* = 1, spine *n* = 1, suprarenal mass *n* = 1 and thyroid *n* = 4	Advanced *n* = 63	WL > 5% from pretreatment weight, BMI < 20 and haemoglobin < 12 g/dL and energy intake < 1500 kcal/day	Not specified	Cachexia *n* = 63	Nutritional counselling + atta flour	Nutritional counselling
Obling et al. [82]	NA	Randomised trial (2 arms)	Denmark (1 centre)	47	66.9 (41.5–88.2)	30 (64%)	Oesophagus *n* = 2, stomach *n* = 9, duodenum *n* = 2, pancreas *n* = 27, bile duct *n* = 2 and colorectum *n* = 5	Advanced *n* = 47	None specified	< 5% *n* = 8, 5%–10% *n* = 10 and 10% *n* = 29	Cachexia *n* = 47	Nutritional counselling + PN	Nutritional counselling
Milla et al. [92]	NA	Randomised trial (2 arms)	Spain (4 centres)	57	64.03^c^	37 (65%)	H&N *n* = 4, upper GI (oesophagus, stomach) *n* = 8, lower GI (small and large intestines) *n* = 9, pancreas *n* = 5, liver *n* = 1, lung *n* = 15, gynaecological *n* = 2, urological *n* = 6 and other *n* = 7	I *n* = 1, II *n* = 9, III *n* = 10 and IV *n* = 25	WL > 5% in last 6 months	Not specified	Cachexia *n* = 57	Nutritional counselling + ONS (enriched)	Nutritional counselling + ONS (standard)

*Note:* Studies were prospective designs unless otherwise stated. Randomised trials were parallel design unless otherwise stated. Age and baseline weight loss of participants were reported as mean ± standard deviation or median (range) unless indicated by the following:

^a^ Median only,

^b^ Mean (range),

^c^ Mean only,

^d^ Median (interquartile range),

^e^ Mean (standard error).

Abbreviations: BMI, body mass index; BW, body weight; CRP, C‐reactive protein; EN, enteral nutrition; ENT, ear, nose and throat; ESPEN, European Society for Clinical Nutrition and Metabolism; FA, fatty acids; GI, gastrointestinal; H&N, head and neck; MA, megestrol acetate; MPA, medroxyprogesterone acetate; MUST, Malnutrition Universal Screening Tool; NIS, nutrition impact symptoms; NRSI, nonrandomised study of intervention; ONS, oral nutritional supplement; PA, physical activity; PN, parenteral nutrition; PS, performance status; SMI, skeletal muscle index; WHO, World Health Organisation; WL, weight loss.

**TABLE A2:** Characteristics of included multicomponent interventions (without published results).

Study	Trial registration number	Study design (*n* of arms)	Country (*n* of centres)	Primary cancer sites	Cancer stages	Cachexia or weight loss screening/classification criteria	Intervention components	Comparator
Protocol papers								
Miura et al. [46]	UMIN000028801	Randomised trial (2 arms)	Japan (15 centres)	Lung and pancreas	Advanced (lung) or inoperable (pancreas)	None specified	Nutritional counselling + ONS + resistance exercise + PA counselling	Standard care
Maeng et al. [47]	NCT04907864	Randomised trial (2 arms)	South Korea (1 centre)	Stomach, colorectum, pancreas, biliary tract and lung	Recurrent or metastatic	Pre‐cachexia: WL ≤ 5% in last 6 months, anorexia and metabolic changes, e.g., glucose intolerance; cachexia: WL > 5% in the last 6 months or BMI < 20 and WL > 2% in the last 6 months	Nutritional counselling + ONS + omega 3 FA + ibuprofen + Bojungikki‐tang + resistance exercise + aerobic exercise + flexibility exercise	Conventional palliative care
Waldman et al. [55]	NCT04629300	Randomised trial (2 arms)	United States (6 centres)	Lung	III or IV	None specified	Managing energy + symptom self‐management + social support + coping skills + reflection	Usual oncology care
Neuzillet et al. [57]	NCT02184663	Randomised trial (2 arms)	France (16 centres)	Pancreas	Locally advanced or metastatic	None specified	Nutritional intervention (oral or EN or PN) as indicated + resistance exercise + aerobic exercise	Usual care
Rogers et al. [66]	ACTRN12611000870954	Randomised trial (2 arms)	New Zealand (1 centre)	Lung	Not specified	Loss of 5% of oedema‐free BW in the last 3–6 months; if no documented WL, BMI < 20.0 kg/m^2^; at least three out of (a) patient‐reported decreased muscle strength, (b) fatigue, (c) patient‐reported anorexia,(d) low fat‐free mass index and (e) abnormal biochemistry	Celecoxib + omega 3 FA + amino acids + resistance exercise	Omega 3 FA + celecoxib
Quist et al. [70]	NCT01881906	Randomised trial (2 arms)	Denmark (1 centre)	Lung	IIIb or IV	None specified	Resistance exercise + aerobic exercise	Usual care (wait‐list control)
Solheim et al. [49]	NCT02330926	Randomised trial (2 arms)	Europe and Canada (13 centres)	Lung and pancreas	Not specified	None specified	Nutritional counselling + ONS + ibuprofen + omega 3 FA + resistance exercise + aerobic exercise	Standard care (wait‐list control)
Moinuddin et al. [78]	Not specified	Randomised trial (4 arms)	United States (1 centre)	Any	Any	WL ≥ 5% in the last 12 months, plus any three of the following criteria: decreased muscle strength, fatigue, anorexia, low fat‐free mass index and abnormal biochemistry	Citrulline + resistance exercise	Control *OR* resistance exercise only *OR* citrulline only
Trial records								
Li et al. [86]	NCT02681601	Randomised trial (2 arms)	United States	Pancreas	Unresectable	WL > 5% in last 6 months	Diet prescription + ONS + omega 3 FA	Diet prescription
Waqar et al. [85]	NCT01501396	Randomised trial (2 arms)	United States	Not specified	Not specified	WL ≥ 5% in the last 6 weeks or WL ≥ 10% in the last 6 months and poor appetite	MA + mirtazapine	MA
Strasser et al. [84]	NCT01127386	Randomised trial (3 arms)	Switzerland	Any	Advanced (locally recurrent or metastatic) and incurable	Cachexia: WL ≥ 2% in last 2 months or ≥ 5% in last 6 months and ongoing in last 4 weeks and CRP ≥ 30 mg/L	Lenalidomide + prokinetics + nutritional counselling + PA counselling	Prokinetics + nutritional counselling + PA counselling
Dev et al. [83]	NCT00625742	NRSI—before–after study (1 arm)	United States	Not specified	Not specified	WL ≥ 5% in last 6 months	Nutritional counselling + ONS + melatonin + ibuprofen + atenolol if indicated + resistance exercise + aerobic exercise	None (uncontrolled)
Naito et al. [80]	PRN‐jRCTs041210053	Randomised trial (2 arms)	Japan	Lung and pancreas	Locally advanced or metastatic	Cancer cachexia with an indication of anamorelin hydrochloride	Nutritional counselling + anamorelin + resistance exercise + PA counselling	Active control
Croagh et al. [91]	ACTRN12624000084583	Randomised trial (2 arms)	Australia	Pancreas	Borderline resectable, locally advanced or metastatic	None specified	Nutritional counselling + education + ONS + EN if indicated + pharmacological NIS management	Usual nutrition care
Yen et al. [90]	NCT05915325	NRSI—before–after study (1 arm)	Taiwan	Not specified	Not specified	Pre‐cachexia: WL 1%–5% in last 6 months or WL 1%–2% and BMI < 20 and one of: elevated CRP, impaired fasting glucose or diabetes, corticosteroid use, hypogonadism and insufficient calorie intake (< 20 kcal/kg/day); cachexia: WL > 5% in last 6 months or WL > 2% and BMI < 20	Nutritional counselling + aerobic exercise + resistance exercise	None (uncontrolled)
van Laarhoven et al. [89]	NCT06138223	Randomised trial (2 arms)	Netherlands	Oesophagus and stomach	Incurable	None specified	Nutritional counselling + aerobic exercise + resistance exercise + PA counselling	Usual care
Peyrachon et al. [88]	NCT06323733	NRSI—before–after study (1 arm)	France	Not specified	Not specified	Cachexia according to Martin et al. classification	Aerobic exercise + resistance exercise	None (uncontrolled)
Unspecified et al. [87]	NCT06338683	Randomised trial (2 arms)	China	Stomach, oesophagus, hepato‐pancreaticobiliary and lung	III or IV inoperable or metastatic	None specified	Nutritional counselling + olanzapine	Nutritional counselling

*Note:* Studies were all prospective design. Randomised trials were parallel design unless otherwise stated. For trial records, the name is the principal investigator and the year is when the record was first posted on the trial register.

Abbreviations: BMI, body mass index; BW, body weight; CRP, C‐reactive protein; EN, enteral nutrition; FA, fatty acids; MA, megestrol acetate; NIS, nutrition impact symptoms; NRSI, nonrandomised study of intervention; ONS, oral nutritional supplement; PA, physical activity; PN, parenteral nutrition; WL, weight loss.
