# Supplementary material for: Multicomponent Interventions for Adults With Cancer Cachexia: A Systematic Review
Source: J Cachexia Sarcopenia Muscle. 2025 Feb 27;16(2):e13716. doi: 10.1002/jcsm.13716 (PMC11865637; doi:10.1002/jcsm.13716)
Supplement: Supplementary file 1 — Table S1.1 Search strategy for MEDLINE via Ovid. Table S1.2 Search strategy for EMBASE via Ovid. Table S1.3 Search strategy for CINAHL via EBSCO. Table S1.4 Search strategy for Cochrane Central Register of Controlled Trials. Table S1.5 Search strategy for ClinicalTrials.gov. Table S1.6 Search strategy for WHO ICTRP. Table S1.7 Search strategy for MedRxiv. [file JCSM-16-e13716-s002.docx]

**Supporting Information 1**

This supporting information file contains the full search strategies for the four databases, two clinical trial registers, and MedRxiv.

**Table S1.1** Search strategy for MEDLINE via Ovid

| # | Query |
| --- | --- |
| 1 | exp Neoplasms/ |
| 2 | Cancer*.ti,ab. |
| 3 | Neoplas*.ti,ab. |
| 4 | Malignan*.ti,ab. |
| 5 | Tumo?r*.ti,ab. |
| 6 | Carcinoma*.ti,ab. |
| 7 | Melanoma*.ti,ab. |
| 8 | Sarcoma*.ti,ab. |
| 9 | Lymphoma*.ti,ab. |
| 10 | Leuk?emia*.ti,ab. |
| 11 | Metasta*.ti,ab. |
| 12 | Adenocarcinoma*.ti,ab. |
| 13 | Choriocarcinoma*.ti,ab. |
| 14 | Teratoma*.ti,ab. |
| 15 | Oncolog*.ti,ab. |
| 16 | exp Cachexia/ |
| 17 | Cachexi*.ti,ab. |
| 18 | Cachectic*.ti,ab. |
| 19 | Anorexi*.ti,ab. |
| 20 | (Weight* adj2 (los* or reduc* or gain* or change*)).ti,ab. |
| 21 | (Wasting* or wasted*).ti,ab. |
| 22 | (Malnutrition* or malnourish*).ti,ab. |
| 23 | Underweight*.ti,ab. |
| 24 | exp Malnutrition/ |
| 25 | exp Wasting Disease, Chronic/ or exp Wasting Syndrome/ |
| 26 | (cancer* adj1 (palliative* or terminal* or incurable* or advanced*)).ti,ab. |
| 27 | ((multi?modal* or multi?component* or bi?modal* or multi?disciplinary* or inter?disciplinary* or combin* or concurrent* or multi?target* or multi?factorial* or rehabilit or pharmacological* or non?pharmacological*) adj3 (interven* or treat* or manage* or approach* or care* or clinic* or regime* or program* or rehabilit*)).mp. |
| 28 | or/1-15 |
| 29 | or/16-26 |
| 30 | 27 and 28 and 29 |

**Table S1.2** Search strategy for EMBASE via Ovid

| 1 | exp malignant neoplasm/ |
| --- | --- |
| 2 | Cancer*.ti,ab. |
| 3 | Neoplas*.ti,ab. |
| 4 | Malignan*.ti,ab. |
| 5 | Tumo?r*.ti,ab. |
| 6 | Carcinoma*.ti,ab. |
| 7 | Melanoma*.ti,ab. |
| 8 | Sarcoma*.ti,ab. |
| 9 | Lymphoma*.ti,ab. |
| 10 | Leuk?emia*.ti,ab. |
| 11 | Metasta*.ti,ab. |
| 12 | Adenocarcinoma*.ti,ab. |
| 13 | Choriocarcinoma*.ti,ab. |
| 14 | Teratoma*.ti,ab. |
| 15 | Oncolog*.ti,ab. |
| 16 | exp cachexia/ |
| 17 | Cachexi*.ti,ab. |
| 18 | Cachectic*.ti,ab. |
| 19 | Anorexi*.ti,ab. |
| 20 | (Weight* adj2 (los* or reduc* or gain* or change*)).ti,ab. |
| 21 | (Wasting* or wasted*).ti,ab. |
| 22 | ((multi?modal* or multi?component* or bi?modal* or multi?disciplinary* or inter?disciplinary* or combin* or concurrent* or multi?target* or multi?factorial* or rehabilit* or pharmacological* or non?pharmacological*) adj3 (interven* or treat* or manage* or approach* or care* or clinic* or regime* or program* or rehabilit*)).mp. |
| 23 | or/1-15 |
| 24 | or/16-21 |
| 25 | 22 and 23 and 24 |

**Note**: due to the high volume of results retrieved in EMBASE, the cachexia terms were reduced for this database.

**Table S1.3** Search strategy for CINAHL via EBSCO

| **#** | **Query** |
| --- | --- |
| S1 | (MH "Neoplasms+") |
| S2 | TI cancer* or AB cancer* |
| S3 | TI neoplas* or AB neoplas* |
| S4 | TI malignan* or AB malignan* |
| S5 | TI tumor* or TI tumour* or AB tumor* or AB tumour* |
| S6 | TI carcinoma* OR AB carcinoma* |
| S7 | TI melanoma* or AB melanoma* |
| S8 | TI sarcoma* or AB sarcoma* |
| S9 | TI lymphoma* or AB lymphoma* |
| S10 | TI (leukemia* or leukaemia*) or AB (leukemia* or leukaemia*) |
| S11 | TI metasta* or AB metasta* |
| S12 | TI adenocarcinoma* or AB adenocarcinoma* |
| S13 | TI choriocarcinoma* or AB choriocarcinoma* |
| S14 | TI teratoma* or AB teratoma* |
| S15 | TI oncolog* or AB oncolog* |
| S16 | (MH "Cachexia") |
| S17 | TI cachexi* or AB cachexi* |
| S18 | TI cachectic* or AB cachectic* |
| S19 | TI anorexi* or AB anorexi* |
| S20 | TI (weight* N2 (los* or reduc* or gain* or change*)) or AB (weight* N2 (los* or reduc* or gain* or change*)) |
| S21 | TI (wasting* or wasted*) or AB (wasting* or wasted*) |
| S22 | TI (malnutrition* or malnourish*) or AB (malnutrition* or malnourish*) |
| S23 | TI underweight* or AB underweight* |
| S24 | (MH "Malnutrition+") |
| S25 | (MH "Wasting Syndrome") |
| S26 | TI (cancer* N1 (palliative* or terminal* or incurable* or advanced*)) or AB (cancer* N1 (palliative* or terminal* or incurable* or advanced*)) |
| S27 | TX ((multi-modal* or multimodal* or multi-component* or multicomponent* or bi-modal* or bimodal* or multi-disciplinary* multidisciplinary* or inter-disciplinary* or interdisciplinary* or combin* or concurrent* or multitarget* or multi-target* or multifactorial* or multi-factorial* or rehabilit* or pharmacological* or nonpharmacological* or non-pharmacological*) N4 (interven* or treat* or manage* or approach* or care* or clinic* or regime* or program* or rehabilit*) |
| S28 | S1 OR S2 OR S3 OR S4 OR S5 OR S6 OR S7 OR S8 OR S9 OR S10 OR S11 OR S12 OR S13 OR S14 OR S15 |
| S29 | S16 OR S17 OR S18 OR S19 OR S20 OR S21 OR S22 OR S23 OR S24 OR S25 OR S26 |
| S30 | S27 AND S28 AND S29 |

**Table S1.4** Search strategy for Cochrane Central Register of Controlled Trials

| # | Query |
| --- | --- |
| #1 | MeSH descriptor: [Neoplasms] explode all trees |
| #2 | (cancer*) |
| #3 | (neoplas*) |
| #4 | (malignan*) |
| #5 | (tumor* or tumour*) |
| #6 | Carcinoma*) |
| #7 | Melanoma*) |
| #8 | Sarcoma*) |
| #9 | Lymphoma*) |
| #10 | Leukemia* or leukaemia*) |
| #11 | Metasta*) |
| #12 | Adenocarcinoma*) |
| #13 | Choriocarcinoma*) |
| #14 | Teratoma*) |
| #15 | Oncolog*) |
| #16 | MeSH descriptor: [Cachexia] explode all trees |
| #17 | Cachexi*) |
| #18 | Cachectic*) |
| #19 | Anorexi*) |
| #20 | (Weight* near/2 (los* or reduc* or gain* or change*)) |
| #21 | (Wasting* or wasted*) |
| #22 | (Malnutrition* or malnourish*) |
| #23 | Underweight* |
| #24 | MeSH descriptor: [Malnutrition] explode all trees |
| #25 | MeSH descriptor: [Wasting Syndrome] this term only |
| #26 | (cancer* near/1 (palliative* or terminal* or incurable* or advanced*)).ti,ab. |
| #27 | ((multi-modal* or multimodal* or multi-component* or multicomponent* or bi-modal* or bimodal* or multi-disciplinary* multidisciplinary* or inter-disciplinary* or interdisciplinary* or combin* or concurrent* or multitarget* or multi-target* or multifactorial* or multi-factorial* or rehabilit* or pharmacological* or nonpharmacological* or non-pharmacological*) near/3 (interven* or treat* or manage* or approach* or care* or clinic* or regime* or program* or rehabilit*)) |
| #28 | #1 or #2 or #3 or #4 or #5 or #6 or #7 or #8 or #9 or #10 or #11 or #12 or #13 or #14 or #15 |
| #29 | #16 or #17 or #18 or #19 or #20 or #21 or #22 or #23 or #24 or #25 or #26 |
| #30 | #27 and #28 and #29 |

**Table S1.5** Search strategy for Clinicaltrials.gov

| Condition or disease | Cancer OR carcinoma OR melanoma OR sarcoma OR lymphoma OR leukaemia |
| --- | --- |
| Other terms | Cachexia OR cachexic OR cachectic OR anorexia OR anorexic OR "weight loss" OR "weight reduction" OR wasting OR wasted OR malnutrition OR malnourished OR underweight |
| Intervention/treatment | Intervention OR treatment OR management OR manage OR approach OR care OR clinic OR regimen OR regime OR program OR programme OR rehabilitation |

**Table S1.6** Search strategy for WHO ICTRP

| Condition | (cancer OR neoplasm OR malignancy OR tumour OR tumor OR carcinoma OR melanoma OR sarcoma OR lymphoma OR leukaemia or leukemia) AND (cachexia OR cachexic OR cachectic OR anorexia OR anorexic OR weight loss OR weight reduction OR wasting OR wasted OR malnutrition OR malnourished OR underweight) |
| --- | --- |

**Table S1.7** Search strategy for MedRxiv

| Search terms & keywords | (cancer*) and (cachexi* or cachectic* or anorexi*) and (interven*) |
| --- | --- |
