# Supplementary material for: Multicomponent Interventions for Adults With Cancer Cachexia: A Systematic Review
Source: J Cachexia Sarcopenia Muscle. 2025 Feb 27;16(2):e13716. doi: 10.1002/jcsm.13716 (PMC11865637; doi:10.1002/jcsm.13716)
Supplement: Supplementary file 2 — Data S1 Supporting Information. [file JCSM-16-e13716-s001.docx]

**Supporting Information 2**

This supporting information file contains equations and additional information on the methods used to calculate the effect of interventions on quality of life over time.

**Mean change in quality of life score over time**

If the mean change in quality of life score was not reported in the study, but the mean baseline quality of life score and the mean post-intervention quality of life score were reported, then we calculated the mean change in quality of life score, using the equation below.

*Mean change score = mean post-intervention score – mean baseline score*

We used a standardised version of the mean change in quality of life score (termed standardised mean change; SMC), using the equation below.

*SMC = mean change score / standard deviation*

**Difference in mean change in quality of life scores between arms**

If the mean difference was not reported in the study, we calculated it using the equation below.

*Mean difference = mean change in quality of life score in multi-component intervention arm – mean change in quality of life score in usual care arm*

We used a standardised version of the mean difference (termed standardised mean difference; SMD), specifically, the adjusted Hedges *g* version of SMD. For our analysis, SPSS calculated the adjusted Hedges *g* and its 95% confidence intervals based on mean change in quality of life score and standard error.

**Standard deviation**

If a study did not report standard deviation, then we calculated or estimated it, where possible. If within-group standard error was reported, we calculated the within-group standard deviation using the formula below.

$$SD=SE \times\sqrt{n}$$

If neither standard error nor standard deviation were reported, but the *p*-value of a *t*-test was reported and the MD was available (either reported or calculated), then we used the method below to calculate the average within-group standard deviation, which we then used for both arms.

*1: to calculate degrees of freedom:*

$$n\left( experimental \right)+n\left( control \right)-2$$

*2: to calculate* t*-statistic in Excel: = tinv(p, degrees of freedom)*

*3: to calculate standard error:*

$$SE = \frac{MD}{t}$$

*4: to calculate average within-group standard deviation:*

$$SD = \frac{SE}{\sqrt{\frac{1}{n(experimental)}+\frac{1}{n(control)}}}$$
